# A Clinically Applicable Positive Allosteric Modulator of GABA Receptors Promotes Human *β*-Cell Replication and Survival as well as GABA's Ability to Inhibit Inflammatory T Cells

**DOI:** 10.1155/2019/5783545

**Published:** 2019-02-26

**Authors:** Jide Tian, Hoa Dang, Nataliya Karashchuk, Irvin Xu, Daniel L. Kaufman

**Affiliations:** Department of Molecular and Medical Pharmacology, University of California, Los Angeles, CA, USA

## Abstract

A major goal of T1D research is to develop new approaches to increase *β*-cell mass and control autoreactive T cell responses. GABA_A_-receptors (GABA_A_-Rs) are promising drug targets in both those regards due to their abilities to promote *β*-cell replication and survival, as well as inhibit autoreactive T cell responses. We previously showed that positive allosteric modulators (PAMs) of GABA_A_-Rs could promote rat *β*-cell line INS-1 and human islet cell replication *in vitro*. Here, we assessed whether treatment with alprazolam, a widely prescribed GABA_A_-R PAM, could promote *β*-cell survival and replication in human islets after implantation into NOD/scid mice. We observed that alprazolam treatment significantly reduced human islet cell apoptosis following transplantation and increased *β*-cell replication in the xenografts. Evidently, the GABA_A_-R PAM works in conjunction with GABA secreted from *β*-cells to increase *β*-cell survival and replication. Treatment with both the PAM and GABA further enhanced human *β*-cell replication. Alprazolam also augmented the ability of suboptimal doses of GABA to inhibit antigen-specific T cell responses *in vitro*. Thus, combined GABA_A_-R agonist and PAM treatment may help control inflammatory immune responses using reduced drug dosages. Together, these findings suggest that GABA_A_-R PAMs represent a promising drug class for safely modulating islet cells toward beneficial outcomes to help prevent or reverse T1D and, together with a GABA_A_-R agonist, may have broader applications for ameliorating other disorders in which inflammation contributes to the disease process.

## 1. Introduction

Clinical trials of interventive therapies for type 1 diabetes (T1D) have not been able to preserve insulin production over the long term such that it is now widely believed that combination treatments that can both control autoimmunity and increase *β*-cell mass will be necessary for effective T1D intervention [1, 2]. GABA receptors (GABA-Rs) are promising targets for next-generation T1D therapies because of their desirable effects on both islet cells and immune cells. In the islets, *β*-cells express both GABA_A_-Rs and GABA_B_-Rs and their activation has been shown to promote *β*-cell survival, replication, and mass [3–8]. Notably, after implanting a minimal mass of human islets into immune-deficient mice, the ability of GABA treatment to promote *β*-cell replication did not attenuate over a five-week observation period leading to increased *β*-cell mass and function [7].

In the immune system, rodent and human T cells express GABA_A_-Rs [6, 9–11]. GABA-R activation has been shown to inhibit or reverse disease in animal models of type 1 diabetes (T1D) [4, 6, 9, 12–14], rheumatoid arthritis [15], and experimental autoimmune encephalomyelitis [16, 17] and to limit inflammation and disease in type 2 diabetes models [18–20]. Studies of GABA's protective mechanisms in these disease models revealed that GABA inhibits inflammatory Th1, Th17, and CD8^+^ responses [4, 6, 9, 13–15], as well as antigen-presenting cell function and TNF*α* production [16, 21, 22]. Thus, GABA-R activation can inhibit inflammation caused by different mechanisms in mice with different genetic backgrounds.

GABA_A_-Rs play a prominent role in regulating neuronal activity, and pharmacological modulation of GABA_A_-Rs is often the primary approach for treating a number of neurological and neuropsychological disorders including seizure, anxiety, and insomnia [23, 24]. Because orally administered GABA has little to no ability to pass through the blood-brain barrier (BBB), BBB-permeable GABA_A_-R PAMs, such as the benzodiazepine alprazolam, have been widely used to enhance the action of GABA secreted by neurons in the central nervous system (CNS). These PAMs do not bind to the GABA-binding site but rather elsewhere on GABA_A_-Rs and increase Cl^−^ conductance when GABA is bound to the receptors [23, 24].

Since islet *β*-cells express, synthesize, and secrete GABA, an administered GABA_A_-R PAM may work in conjunction with GABA secreted by *β*-cells in an autocrine fashion to promote *β*-cell survival and mitogenesis. Indeed, we previously showed that some GABA_A_-R PAMs, such as alprazolam, were capable of promoting the replication of rat INS-1 *β*-cells or human islet cells *in vitro* without the addition of exogenous GABA [25]. These effects were blocked by the GABA_A_-R antagonist bicuculline [25]. Here, we examined whether administering a GABA_A_-R PAM could promote human *β*-cell replication and survival *in vivo* using a human islet xenograft model. We focused on testing alprazolam because (1) it is widely prescribed for treating anxiety, (2) it does not have off-target effects on the “peripheral benzodiazepine receptor” (now known to be a mitochondrial translocator protein [26]), and (3) it is safe for long-term use when used as directed [27, 28]. Finally, we examined whether GABA_A_-R PAMs also have potential for helping GABA to inhibit inflammatory T cell responses. Our results suggest that GABA_A_-R PAMs may be a new drug class to safely help in diabetes prevention and treatment.

## 2. Materials and Methods

### 2.1. Chemicals

Alprazolam, *streptozotocin* (*STZ*), and 5-bromo-2-deoxyuridine (BrdU) were purchased from Sigma-Aldrich.

### 2.2. Analysis of Human *β*-Cell Apoptosis *In Vivo*

All animal experiments were approved by UCLA's Animal Research and Care Committee. Fresh human islets were obtained from the Integrated Islet Distribution Program (IIDP). All batches of human islets were received at UCLA within 36 hours of isolation from donors < 45 years in age and had >90% viability and >85% purity, which in our experience are optimal human islet parameters for studying *β*-cell replication in this xenograft model. The day before receiving the islets, NOD/scid mice (Taconic Farms) were injected intraperitoneally (IP) with STZ (220 mg/kg) and diabetic mice (blood glucose levels between 220 and 350 mg/dl) were implanted with about 2000 human islet equivalents under their kidney capsule. The following day, the mice were randomized into the following treatment groups: (1) alprazolam IP daily at the indicated dose—the alprazolam was made fresh daily by dissolving it into DMSO and then diluting to 5% DMSO in sterile PBS, (2) vehicle only (5% DMSO in PBS) daily IP, (3) oral GABA (6 mg/ml, continuously through their drinking water), or (4) combined alprazolam (at the indicated dose) and oral GABA. After two days of drug treatment, the engrafted kidneys were harvested, and the percentages of insulin^+^*β*-cells or TUNEL^+^ apoptotic islet cells in total islet cells within the grafts of individual recipients were determined by immunofluorescence in a blinded manner, as in our previous report [5].

### 2.3. Analysis of Human *β*-Cell Replication *In Vivo*

STZ-rendered diabetic NOD/scid mice were implanted with about 2000 human islet equivalents under their kidney capsule. Two days after transplantation, the mice were randomized to receive the following: (1) alprazolam daily IP (at the indicated dose), (2) vehicle (5% DMSO in PBS) daily IP, (3) oral GABA (6 mg/ml, continuously through their drinking water), or (4) combined alprazolam (at the indicated dose) and GABA. The drinking water of all groups contained BrdU. After ten days of treatment, their kidney tissue sections were subjected to immunofluorescent analysis [5]. Briefly, the islet graft sections were stained with PE-anti-human Ki67 (eBioscience), guinea pig anti-insulin, or biotinylated anti-BrdU (AbD Serotec), and after being washed, the sections were reacted with FITC-anti-guinea pig IgG and PE-streptavidin, followed by counterstaining with DAPI. The percentages of BrdU^+^insulin^+^ and Ki67^+^insulin^+^*β*-cells in at least 2000 islet cells of 10 fields (magnification ×400) of each islet graft were analyzed in a blinded manner [5].

### 2.4. T Cell Proliferation Assay

C57BL/6 mice were injected with hen egg lysozyme (HEL, a prototypic foreign antigen) in 50% complete Freund's adjuvant (CFA) in their hind footpad. Nine days later, their lymph node mononuclear cells were isolated and 3 × 10^5^ mononuclear cells/well were tested in triplicate in 1% FCS HL-1 medium for T cell recall responses to HEL in the presence or absence of different concentrations of alprazolam and/or GABA. During the last 16 h of a 96-hour incubation period, 1 *μ*Ci ^3^H thymidine was added into each well. The thymidine uptake in individual wells of cells was measured using a *β*-counter.

### 2.5. Statistical Analysis

Group sizes and the number of biological replicates are detailed in each figure legend. Data are expressed as the mean ± SEM of individual groups. The difference among groups was analyzed by ANOVA and post hoc Fisher's least significant difference, and the difference between groups was determined by a Student *t*-test. A two-tailed *p* value of <0.05 was considered statistically significant.

## 3. Results

### 3.1. A GABA_A_-R PAM Improves Human Islet Cell Survival, and to a Greater Extent When Combined with Exogenous GABA Treatment, following Islet Transplantation

A major difficulty in human islet transplantation arises from the apoptosis of a large proportion of islet cells within a few days following implantation [29, 30]. We and others have demonstrated that GABA treatment can promote human islet cell survival following transplantation [5–7, 14, 31]. Here, we asked whether a GABA_A_-R PAM, in the absence of GABA administration, could limit islet cell apoptosis *in vivo* using a human islet xenograft model.

**Figure 1:**
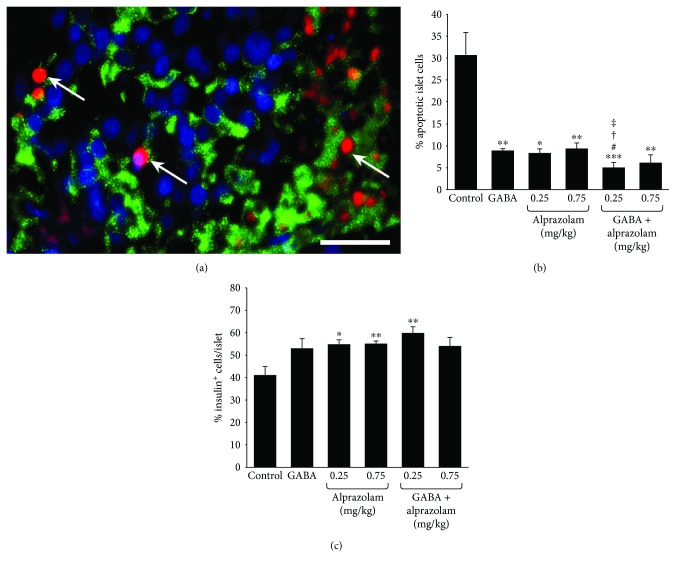
Alprazolam reduces islet cell apoptosis in human islet xenografts. Mice receiving human islet grafts were treated IP with vehicle or alprazolam (0.25 or 0.75 mg/kg/day IP) and/or GABA through their drinking water (6 mg/ml) or water alone. The frequency of apoptotic cells in the different groups of recipients was determined by TUNEL and anti-insulin staining assays. All control and experimental groups were tested side by side. Data are a representative image (magnification ×400) or expressed as the mean % of apoptotic islet cells ± SEM of each group (*n* = 4-6 mice) from four islet donors in four separate experiments (with one islet donor for each experiment). (a) A representative image of apoptotic islet cells (red) and insulin^+^*β*-cells (green) in human islet grafts. Scale bar = 25 *μ*m. (b) Frequency of apoptotic cells. (c) Frequency of insulin^+^*β*-cells. White arrows indicate apoptotic cells. ^∗^*p* < 0.05, ^∗∗^*p* < 0.01, and ^∗∗∗^*p* < 0.001 vs. the control with vehicle injection and plain water. ^#^*p* < 0.05 vs. GABA treated. ^†^*p* < 0.05 vs. the alprazolam (0.25 mg/kg/day) and ^‡^*p* < 0.05 vs. the alprazolam (0.75 mg/kg/day).

NOD/scid mice were STZ-rendered diabetic and implanted with human islets under their kidney capsule. The next day, the mice were randomized and treated IP with alprazolam daily at the indicated dose or vehicle (negative control). Another group of mice received only GABA (6 mg/ml, positive control) continuously through their drinking water. All of the implanted mice became normoglycemic within two days after receiving the islet graft. After two days of treatment, the implanted kidneys were removed and kidney tissue sections were stained by TUNEL and anti-insulin antibodies (Figure 1(a)). We observed that treatment with GABA reduced the number of TUNEL^+^ islet cells to only 25% of that observed in the islet xenografts of mice that received vehicle alone (Figure 1(b)) consistent with previous observations [5, 7]. Treatment with alprazolam at each of the tested dosages similarly significantly reduced the frequency of apoptotic islet cells, relative to that in the control group (Figure 1(b)). Notably, the combination of GABA and alprazolam (at 0.25 mg/kg/day) treatment further decreased the percentages of apoptotic islet cells, relative to either monotherapy. Along with decreased islet cell apoptosis, we observed that alprazolam treatment (at both 0.25 and 0.75 mg/kg) significantly increased the percentage of insulin^+^*β*-cells (Figure 1(c)). GABA treatment tended to increase the percentage of insulin^+^*β*-cells (*p* = 0.08). While combined GABA and alprazolam (at 0.25 mg/kg/day, but not at 0.75 mg/kg/day) treatment further increased the average percentages of *β*-cells, this was not statistically significant compared to either monotherapy (Figure 1(c)). Given that alprazolam itself cannot activate GABA_A_-Rs, our observations suggest that alprazolam may work with endogenous GABA secreted by *β*-cells to limit islet cell apoptosis and promote *β*-cell survival.

### 3.2. A GABA_A_-R PAM Promotes Human *β*-Cell Replication *In Vivo*, and to a Greater Extent When Administered Together with GABA

**Figure 2:**
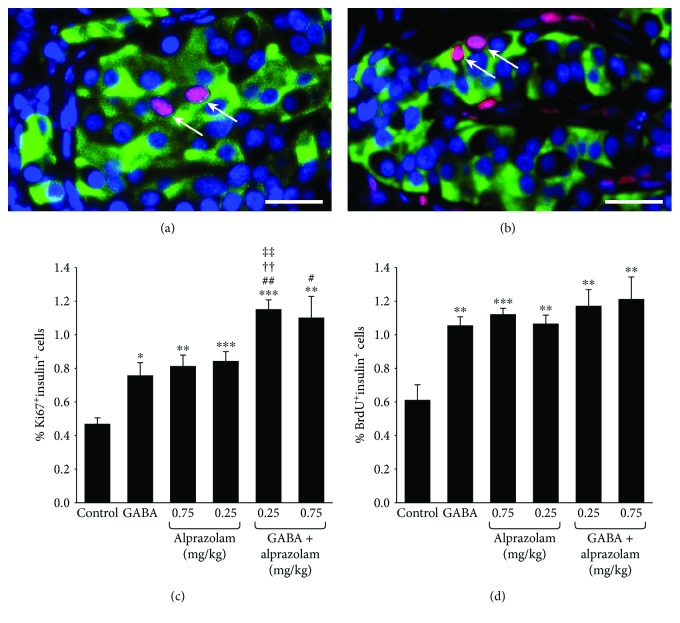
A GABA_A_-R PAM promotes human *β*-cell replication, and to a greater extent when combined with exogenous GABA treatment. Hyperglycemic NOD/scid mice were transplanted with human islets under the kidney capsule. All islet recipients became normoglycemic within two days after transplantation. They were then randomized to receive vehicle or alprazolam (0.25 or 0.75 mg/kg/day IP) and/or GABA through their drinking water (6 mg/ml). All mice received BrdU through their drinking water as described in Materials and Methods. After ten days of drug treatment, the replication of human *β*-cells in the islet grafts was characterized by immunofluorescent staining of Ki67/insulin and BrdU/insulin as described in Materials and Methods. The data are representative images (magnification ×400) or expressed as the mean percentages of Ki67^+^insulin^+^ or BrdU^+^insulin^+^ islet cells ± SEM of each group (*n* = 5-6) of mice from at least three separate experiments. (a) Representative image of islet cells (magnification ×400) costained with anti-insulin (green) and anti-Ki67 (red) (arrows). Scale bar = 25 *μ*m. (b) Representative image of islet cells costained with anti-insulin (green) and anti-BrdU (red) (arrows). (c) The percentages of Ki67^+^insulin^+^ cells in the islet grafts. (d) The percentages of BrdU^+^insulin^+^ cells in the grafts. ^∗^*p* < 0.05, ^∗∗^*p* < 0.01, and ^∗∗∗^*p* < 0.001 vs. the control with vehicle injection and plain water. ^#^*p* < 0.05 and ^##^*p* < 0.01 vs. the GABA-treated mice. ^††^*p* < 0.01 vs. the alprazolam alone (0.25 mg/kg/day). ^‡‡^*p* < 0.01 vs. the alprazolam alone (0.75 mg/kg/day).

We next assessed whether alprazolam could promote human *β*-cell replication in an islet xenograft model. STZ-rendered diabetic NOD/scid mice received human islets under their kidney capsule, and within two days, all of the recipients became normoglycemic. They were then randomized and treated with (1) alprazolam (0.025 or 0.75 mg/kg/day IP), (2) vehicle IP (control), (3) oral GABA, or (4) oral GABA together with alprazolam (0.25 or 0.75 mg/kg daily). All groups received BrdU through their drinking water. After ten days of treatment, the mice were sacrificed and their kidneys were processed for immunofluorescent examination of the islet grafts (Figure 2(a) and 2(b)).

As expected, the frequency of Ki67^+^insulin^+^*β*-cells in the human islet grafts in the GABA-treated mice was about 170% greater than that in control mice that received plain water (Figure 2(c)). Administration of alprazolam, at either dose, also augmented the frequency of *β*-cell replication, to levels similar to that of GABA. GABA and alprazolam (at both dosages) monotherapies also enhanced *β*-cell replication as determined by analysis of BrdU^+^insulin^+^ cells (Figure 2(d)). Notably, combined treatment with both GABA and alprazolam (at both the 0.25 and 0.75 mg/kg doses) significantly enhanced human *β*-cell replication beyond that evoked by GABA alone, as well as alprazolam alone (at the 0.25 mg/kg dose), as measured by anti-Ki67/insulin immunostaining.

### 3.3. A GABA_A_-R PAM in Combination with GABA More Effectively Suppresses Inflammatory T Cell Responses *In Vitro* than GABA Alone

**Figure 3:**
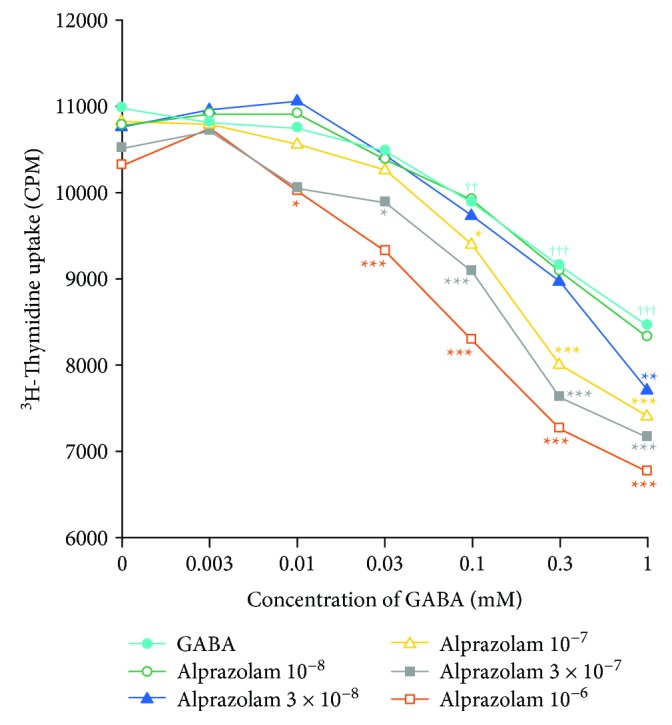
Alprazolam augments the ability of GABA to inhibit antigen-specific T cell proliferative responses. Mice were immunized with HEL, and nine days later, their splenic mononuclear cells were assayed in triplicate for proliferative responses to HEL as described in Materials and Methods. Each line represents a molar concentration of alprazolam together with different concentrations of GABA from six mice in two separate experiments. *p* < 0.05, ^††^*p* < 0.01, and ^†††^*p* < 0.001 vs. the medium alone. ^∗^*p* < 0.05, ^∗∗^*p* < 0.01, and ^∗∗∗^*p* < 0.001 vs. the GABA alone at the same concentration.

Central to the prevention and treatment of T1D is the development of treatments that can downregulate autoreactivity against *β*-cells. Because GABA_A_-R activation can inhibit inflammation and autoimmune diseases in various mouse models, we asked whether a GABA_A_-R PAM may augment this anti-inflammatory activity. C57BL/6 mice were immunized with HEL, a prototypic foreign antigen, and nine days later, their lymph node mononuclear cells were tested *in vitro* for proliferative responses to HEL in the presence or absence of different concentrations of GABA and/or alprazolam. As expected, GABA alone (0.01-1 mM) significantly inhibited HEL-specific T cell proliferation (Figure 3), consistent with our previous work [9]. Notably, alprazolam alone did not significantly change T cell proliferation at any of the dosages tested.

When immune cells were exposed to both alprazolam and GABA, the combination more effectively inhibited T cell proliferation in response to HEL than the same dose of GABA alone. For example, while GABA alone had a significant inhibitory effect beginning at 0.1 mM, when GABA was combined with alprazolam at 1 × 10^−6^ M, the combination significantly reduced T cell proliferation when GABA was present at just 0.01 mM, a tenfold lower level than the lowest effective GABA (alone) dose. It was also notable that GABA (alone) had a significant inhibitory effect at 0.1-1.0 mM and that the inhibitory effect of GABA at 0.1-1 mM was significantly greater at each dose in the presence of alprazolam at 1 × 10^−7^ M and even greater when these dosages of GABA were combined with 3 × 10^−7^ M alprazolam. Thus, alprazolam augmented the inhibitory effect of GABA on antigen-specific T cell proliferation, and their combination had greater inhibitory effect than either drug alone.

## 4. Discussion

GABA_A_-Rs are promising drug targets for helping to prevent or reverse T1D due to their abilities to (1) inhibit inflammatory T cell responses, (2) enhance Treg responses, and (3) promote *β*-cell replication and survival. Discovering approaches to more effectively activate GABA_A_-Rs on *β*-cells and immune cells may therefore have clinical relevance for T1D therapy.

Here, we studied the widely prescribed GABA_A_-R PAM alprazolam. We observed that alprazolam treatment limited the apoptosis of human islet cells that is usually quite extensive shortly after transplantation. In addition, alprazolam increased *β*-cell replication in the xenografts. These observations suggest that including a GABA_A_-R PAM in the drug regimen following clinical human islet transplantation for even a brief period could help limit *β*-cell loss and reduce the number of islets required to achieve insulin independence.

Since alprazolam does not have GABA_A_-R agonist activity, but rather potentiates the action of GABA when GABA is bound to the receptor, our findings suggest that the PAM worked in conjunction with *β*-cell-secreted GABA in an autocrine fashion to increase *β*-cell survival and replication. The amount of GABA in the implanted islet microenvironment, however, appeared to be suboptimal for maximizing *β*-cell replication since combined treatment with exogenous GABA and alprazolam leads to an even greater level of *β*-cell replication. Indeed, in the islet transplant model used here, the islets were implanted into a small area under the kidney capsule such that there was likely to be substantial extracellular GABA in the islet microenvironment. In contrast, in the pancreas of newly diabetic individuals, the islets are dispersed throughout the organ and have lost most of their GABA-producing *β*-cells. In this context, GABA_A_-R PAMS may not effectively promote residual *β*-cell health and replication unless an exogenous GABA_A_-R agonist is supplied.

In the immune system, lymphocytes do not synthesize GABA, but antigen-presenting cells have been reported to synthesize and secrete low levels of GABA [16, 32]. We observed that alprazolam alone had no effect on lymph node T cell responses to an immunogen *in vitro* (Figure 3). Evidently, if the antigen-presenting cells in this assay secreted GABA, it was at an insufficient level to work with alprazolam and induce detectable changes in cellular proliferation. When exogenous GABA was supplied to T cell cultures containing alprazolam, we observed that 10-fold lower levels of GABA effectively inhibited T cell proliferative responses. The general ability of combined GABA and alprazolam to inhibit T cell proliferation and promote *β*-cell survival and replication better than their respective monotherapies suggests that GABA_A_-R-based therapeutics may be effective in the clinic using subclinical dosages of a PAM, such as alprazolam.

The attenuation of GABA synthesis and secretion in the islets due to *β*-cell stress and death during diabetes development is expected to reduce GABA's (1) autocrine effects on *β*-cell replication and survival [3–8], (2) inhibition of glucagon secretion from *α*-cells [33], and (3) regulatory action on inflammatory infiltrates in the islets [12]. Accordingly, the impairment and loss of *β*-cells and the ensuing reduction of secreted GABA may have multiple deleterious consequences that exacerbate the development of hyperglycemia. Administration of a GABA_A_-R agonist could help to restore these activities, and its effects may be enhanced by cotreatment with alprazolam or another GABA_A_-R PAM. Such combined treatments may also help regulate inflammatory T cell responses systemically and help treat a wide range of disorders in which inflammation contributes to the disease process.

## 5. Conclusions

Administration of alprazolam alone effectively limited islet cell apoptosis following human islet transplantation and increased *β*-cell replication in the xenografts. Combined treatment with GABA and alprazolam greatly enhanced human *β*-cell replication in the xenografts. Additionally, treatment with alprazolam augmented the ability of GABA to inhibit inflammatory T cell responses *in vitro*. Thus, combined treatment with a GABA_A_-R agonist and PAM may help promote *β*-cell survival and replication, as well as control inflammatory immune responses using reduced drug dosages.

## Data Availability

The data used to support the findings of this study are available from the corresponding author upon request.
